# Bisphenol A in Disposable Face Masks: A Novel Human Exposure Pathway and Impact on the Aquatic Environment

**DOI:** 10.1021/acs.chemrestox.4c00535

**Published:** 2025-02-03

**Authors:** Hei-Tak Tse, Chun-Kit Au, Wan Chan

**Affiliations:** Department of Chemistry, The Hong Kong University of Science and Technology, Clear Water Bay, Kowloon, Hong Kong

## Abstract

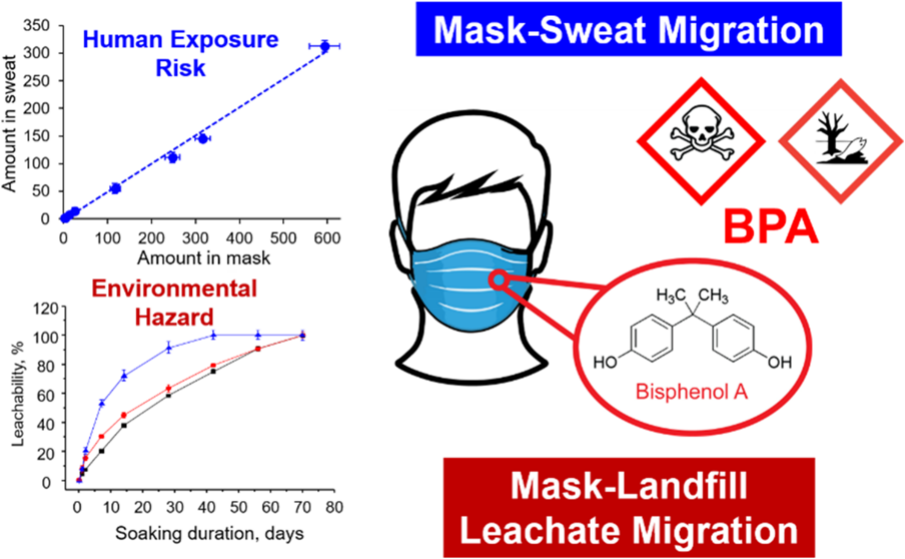

We identified and quantified bisphenol A (BPA), a known estrogen-like endocrine disruptor, in disposable face mask samples collected in Hong Kong. Results revealed that BPA is a common contaminant in face masks, with concentrations reaching up to 2 μg/mask. Although polypropylene, the primary material used in mask production, is generally considered to be BPA-free, the contaminant likely originates from additives, such as flame retardants, added during manufacturing. With a dermal absorption coefficient of 0.59 for BPA, the data indicate that mask-borne BPA is readily absorbed by the skin. Notably, 8 of 85 samples could cause the user to exceed the tolerable daily BPA intake set by the European Food Safety Agency (0.0002 μg/kg body weight per day). Additionally, BPA dissolves completely in landfill leachate in less than 70 days, which poses previously unrecognized health and environmental hazards. Given the extensive use of face masks during the pandemic, their role as personal protective equipment for medical practitioners, and the fact that there are currently no regulations regarding BPA contents in masks, it is imperative to investigate the need for regulations in order to safeguard face mask users and the environment.

## Introduction

Disposable face masks are personal protective equipment widely used in the workplace and in daily life to reduce human exposure to pathogens and airborne particulates, such as the influenza virus and pollen.^1^ During the COVID-19 pandemic, the use of face masks increased exponentially, with over 95% of the world’s population recommended or mandated to wear masks in public areas and an estimated 4 billion masks consumed daily.^2^

Face masks are manufactured from fossil fuel-derived petrochemicals, such as polypropylene, which may contain impurities and additives that negatively impact users’ health when inhaled or through skin contact, especially with routine daily use.^3^ We recently reported the detection of a broad spectrum of semivolatile and volatile organic compounds (VOCs) in face masks, including polycyclic aromatic hydrocarbons (PAHs), phthalate esters, and reactive carbonyls.^4^ Notably, naphthalene and acrolein were found in most of the mask samples, while di(2-ethylhexyl) phthalate, an androgen antagonist, was detected in more than one-third of the samples.

To explore the potential health risks associated with mask-wearing, we expanded our study to quantify bisphenol A (BPA), a well-known estrogen-like endocrine disruptor,^5^ in disposable face masks. While polypropylene, the primary material used in mask production,^5−7^ is generally considered BPA-free, we chose to investigate BPA due to emerging evidence suggesting that it may be a contaminant in face masks. For instance, BPA is utilized in manufacturing various flame retardants for polypropylene, including tetrabromobisphenol A (TBBPA).^8−10^ This finding underscores the importance of examining the safety implications of widespread mask use, especially given the increasing reliance on disposable masks in various settings, e.g., hospitals.

Moreover, with estimates indicating that over 80% of used face masks ultimately end up in landfills,^11^ we also investigated the potential for BPA contaminants to be released into the environment. This aspect of our study is critical as it raises concerns about the long-term environmental impact of disposable masks and the associated risks of chemical leaching. By addressing both human health and environmental dimensions, we aim to provide a comprehensive understanding of the implications of mask usage in our current context.

Using liquid chromatography-tandem mass spectrometry (LC–MS/MS) combined with a stable isotope-dilution method, we analyzed 85 commercially available face masks in Hong Kong for the presence of BPA and its associated analogues. Our findings revealed that BPA was detected in the majority of the mask samples, with the highest concentration detected in the middle filtering layer. Following this initial detection, we further explored the potential migration of BPA from the masks to human skin and the aquatic environment by soaking them in artificial sweat and landfill leachate, respectively. This study uncovers a previously unrecognized pathway for human exposure to BPA and highlights a new source of BPA contamination in the environment.

## Materials and Methods

### Chemicals and Reagents

All chemicals and reagents were of the highest purity available and were used without further purification unless otherwise stated. Bisphenol A (BPA), bisphenol F (BPF), bisphenol C (BPC), bisphenol AP (BPAP), bisphenol AF (BPAF), tetrabromobisphenol A (TBBPA), and heavy-isotope-labeled internal standard bisphenol A-*d*_16_ (BPA-*d*_16_) were obtained from Sigma-Aldrich (St. Louis, MO). High-purity HPLC-grade acetonitrile, dichloromethane, and methanol were purchased from Tedia (Fairfield, OH). Deionized water was further purified by a Pall Cascada Laboratory water purification system (Port Washington, NY).

### Sample Collection and Preparation

Eighty-five different disposable face masks of different origins were purchased from supermarkets in Hong Kong. Prior to the analysis, ear loops were removed, and the mask was separated into layers. The ear loop and each layer of the mask were weighed separately before being cut into 1 cm^2^ pieces.

Sample preparation was conducted essentially as described previously,^12^ with modification. To minimize bisphenol contamination, glassware was used in the sample preparation. Prior to extraction, 15 ng of BPA-*d*_16_ was spiked to the ear loop and individual mask layer, followed by adding 15 mL of 1 M NaOH solution and heating at 70 °C for 1 h. After cooling to room temperature, 500 μL of sample aliquot was transferred to a 2 mL glass tube, adjusted to pH 2 with 6 M HCl, and extracted three times with 330 μL of DCM. The organic extracts were combined and dried with nitrogen gas. The residue was reconstituted in 100 μL of 75% acetonitrile/water for LC–MS/MS analysis.

### Mask-to-Skin BPA Migration

The inner layer of mask and ear loops from ten different mask samples containing different amounts of BPA were soaked in 20 mL artificial sweat, which was prepared as described previously (Table S1).^13^ After incubating at 37 °C for 4 h, 980 μL of the sample extract was sampled and mixed with 20 μL of BPA-*d*_16_ (50 ng/mL), pH adjusted, and subjected to a similar extraction procedure as stated above.

### Mask-to-Landfill Leachate BPA Migration

Artificial landfill leachate was prepared as reported previously (Table S1).^14^ The effects of leachate temperatures (4, 20, and 37 °C), pH (4.0, 6.0, 8.0, and 10.0), and the ages (young and old) of landfill leachate on the leachability of BPA were investigated. Masks with different BPA contents (599.2 ± 2.1, 54.1 ± 0.2, and 13.3 ± 0.1 ng/mask; one-third of a mask was used for each experiment) were cut into small pieces and immersed in 25 mL of artificial landfill leachate and shaken in an incubator at 100 rpm. On days 0, 1, 2, 7, 14, 28, 42, 56, and 70, 1 mL of the landfill leachate was sampled and extracted as described above.

### LC–MS/MS Analysis

Analysis of bisphenols was conducted on a Waters Acquity UPLC instrument connected to a Waters TQ-XS triple quadrupole system (Milford, MA) equipped with an electrospray ionization (ESI) interface. Ten microliters of the sample was injected onto a Phenomenex Luna C18 column (100 mm × 2.0 mm, 5 μm; Torrance, CA) maintained at 40 °C. Bisphenols were chromatographically separated with a flow rate of 0.3 mL/min with a gradient of 0.01% acetic acid in water (A) and ACN (B): 0–1 min, 5% B; 1–5 min, linearly ramped from 5 to 95% B; and held at 95% B for 2 min before re-equilibration for next injection. The mass spectrometer was set to negative ion mode with optimized ESI parameters: cone gas, 500 L/h; desolvation gas, 1000 L/h; collision gas, 0.12 mL/min; nebulizer gas, 7.0 bar; desolvation temperature, 550 °C; capillary voltage, 1.5 kV; and cone voltage, 25 V. MRM transitions *m*/*z* 227/212, *m*/*z* 199/93, *m*/*z* 255/240, *m*/*z* 289/274, and *m*/*z* 335/265 were used for quantitative analysis of BPA, BPF, BPC, BPAP, and BPAF, respectively, whereas *m*/*z* 227*/*132, *m*/*z* 199/106, *m*/*z* 255/147, *m*/*z* 289/211, and *m*/*z* 335/197 were simultaneously monitored for qualitative analysis. The isotope-labeled internal standard, BPA-*d*_16_, was monitored at *m*/*z* 241/142.

### Calibration Curve, Method Validation, Detection Limits, and Quality Control

Calibration curves for quantitative analysis of bisphenols were constructed by plotting the peak area ratios of individual bisphenols to BPA-*d*_16_ versus the respective concentrations of bisphenols in the working standards (0.5 to 50 μg/L; Table S2). Method accuracy was evaluated by spiking different amounts of bisphenols into a bisphenol-free mask, followed by extraction and the amount of bisphenols recovered by LC–MS/MS analysis as mentioned above (Table S3). Intraday and interday precision of the method were determined by analyzing the samples separately on the same day (*n* = 7) and 7 days within 1 week, respectively. The method detection limit was evaluated as the amount of bisphenols that generated an analytical signal three times the noise (Table S3). Reagent blanks and method blanks were included in every batch of 20 samples, according to EPA QA/G-5.^15^

## Results and Discussion

### Quantitation of BPA in Face Masks

BPA is widely used as a comonomer in the manufacturing of various plastics, including polycarbonates.^16^ Humans are continually exposed to BPA through dietary intake, as it can leach from plastic food packaging and migrate into food.^17−20^ Growing evidence indicates that BPA disrupts hormonal and reproductive systems,^21−24^ prompting multiple regulatory agencies to take action to reduce human exposure. For instance, the U.S. Food and Drug Administration (FDA) has banned the use of BPA in baby bottles and sippy cups.^25^ In 2023, the European Food Safety Agency (EFSA) significantly lowered the tolerable daily intake (TDI) of BPA from 4 μg/kg body weight to an astonishing 0.0002 μg/kg body weight.^26^ These measures reflect an urgent response to the potential risks posed by BPA, highlighting the need for continued vigilance and regulation of consumer products.

Although not yet documented in the literature, emerging evidence suggests that disposable face masks may be produced by using BPA-containing additives. For instance, BPA is utilized in the formulation of various flame retardants for polypropylene, a common material in mask production, including TBBPA.^8−10,27^ For example, although not strongly correlated with the detected BPA concentrations, TBBPA was detected in the collected mask samples at concentrations ranging from 2 to 10 ng/mask (Figure S1). In light of this potential source of contamination, we aimed to investigate the presence of BPA in face mask samples.

To achieve this, we employed an isotope-dilution LC–MS/MS method, which allows for the accurate quantification of BPA levels. This study seeks to illuminate a previously overlooked source of BPA exposure through disposable face masks and explore its implications for human health and safety. By addressing this critical issue, we hope to provide valuable insights into the risks associated with the widespread use of disposable face masks. Understanding the presence of BPA in face masks is essential for assessing their safety and ensuring that consumer health is prioritized in the ongoing use of personal protective equipment.

**Figure 1:**
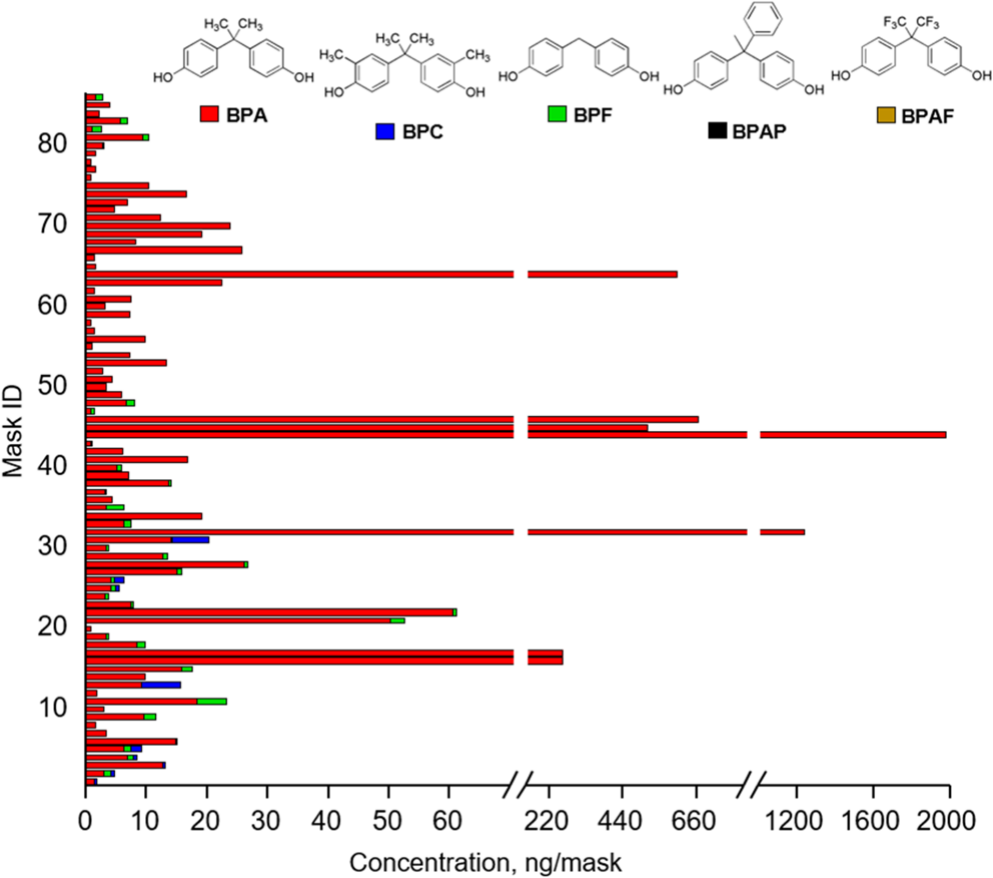
Amount of BPA and its associated analogues in disposable face masks. Shown in the insets are the chemical structures of BPA and its analogues targeted in this study.

To our surprise, the analysis revealed that BPA was present in most of the collected samples, with concentrations ranging from ng to μg/mask in the masks (Figure 1). Shown in Figure S2 is a typical chromatogram obtained from the LC–MS/MS analysis of one of the mask samples. Notably, the mass concentration of BPA varied across different layers of the masks, with the highest concentrations found in the middle filtering layer (Figure S3). While nonwoven fabrics are commonly used for the inner and outer layers of masks, melt-blown fabric is typically employed for the middle filtering layer.^28^ It is likely that various mask manufacturers utilized melt-blown and nonwoven fleece from different sources that were added with different kinds and amounts of additives for the different layers,^28−31^ resulting in the observed range of BPA concentrations. This study marks the first documented detection of BPA in face mask samples, highlighting a significant concern regarding potential contamination.

During the investigation, we also attempted to detect BPC, BPF, BPAF, and BPAP, which are BPA analogues that are often used as less toxic substitutes for BPA.^22^ However, these compounds were detected in only a few samples and at considerably lower concentrations (Figure 1). This suggests that these substitutes were not commonly used as additives during the production of the nonwoven and melt-blown fleece materials employed in mask manufacturing.

### Mask-to-Skin Migration of BPA

The identification of BPA in masks potentially highlighted a previously unrecognized risk of human exposure, particularly during the pandemic and among individuals who wear face masks for occupational purposes. Given the low volatility of BPA (boiling point of around 220 °C), it is less likely to vaporize and be inhaled by mask wearers; therefore, skin absorption of BPA from masks may be a critical factor in assessing this exposure risk. To assess the potential human BPA exposure via mask-wearing, we studied the migration of BPA from masks to the skin by soaking the inner layer and ear loops in artificial sweat, a medium commonly used to examine the migration of chemicals from textiles to skin.^13,32^ These components were selected for testing due to their close contact with the user.

**Figure 2:**
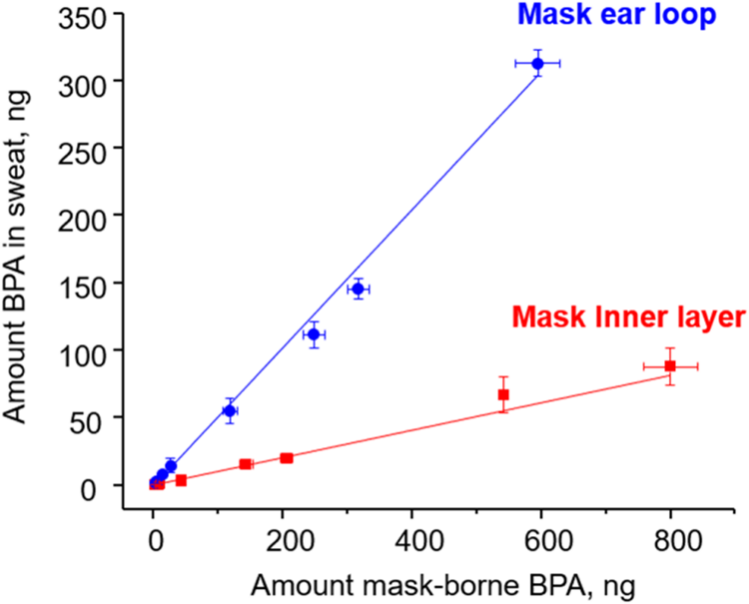
Amount of BPA migrated to sweat after 4 h of soaking in artificial sweat at 37 °C. Mask inner layers (red line) and ear loops (blue line) containing different amounts of BPA were soaked separately in artificial sweat, as described in the Materials and Methods section. The data represent the mean ± SD for three independent experiments. Fitting the data by linear regression yielded lines with the following equations: *y* = 0.11*x*–0.42 (*r*^2^ = 0.99; mask inner layer); *y* = 0.51*x*–2.1 (*r*^2^ = 0.99; mask ear loop).

After 4 h of soaking in artificial sweat at 37 °C, we found that the amount of BPA in the sweat is proportional to the BPA concentration in the mask materials (Figure 2). Specifically, 11 ± 2% and 51 ± 6% of BPA migrated from the inner mask layer and ear loop, respectively. The differing migration rates can likely be attributed to the materials used in manufacturing these components. While polypropylene is the primary material of the inner layer of the face mask, the ear loops are made of polyester.^28,33^ The interaction between the nonpolar BPA and polypropylene is stronger than that with polyester, which is more polar than polypropylene, which may explain the lower migration rate of BPA from the fleece of the inner layer compared to the ear loops.

**Figure 3:**
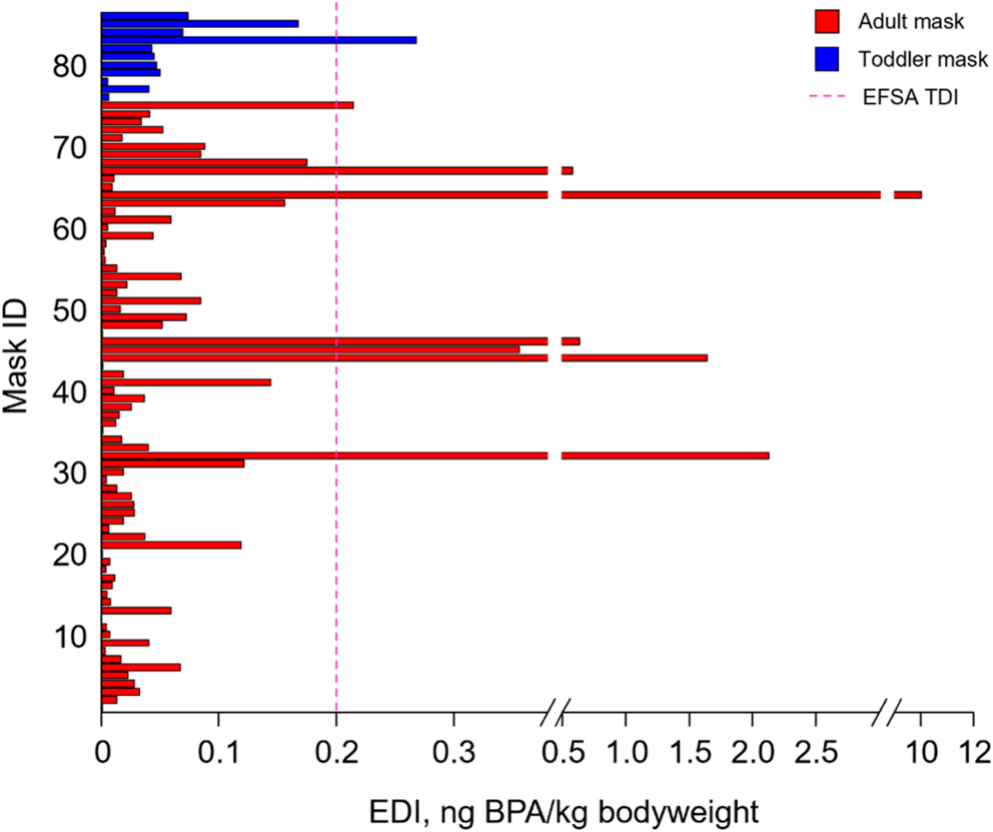
Estimated daily intakes (EDI) of BPA absorbed through dermal contact with face masks, calculated based on the following parameters: two masks used per day, 90% mask-to-face contact surface area, migration rates of 11% from the inner mask layer and 51% from the ear loop, a dermal absorption efficiency of 59%,^34^ body weights: adult 60 kg; toddler 8 kg. The dotted line marked the EFSA BPA exposure limit of 0.0002 μg/kg of body weight per day.

Using a mask-to-face contact surface of 90%,^11^ along with the calculated mask-to-sweat transfer factors, a dermal absorption coefficient of BPA of 0.59,^34^ and accounting for each person using two masks per day (4 h per mask), we estimated the daily BPA exposure dosage from face mask-wearing, as detailed in the Materials and Methods section. The results indicated that under these criteria, 8 out of the 85 mask samples (Figure 3 and Table S4) may have caused the user to exceed the EFSA limit on BPA exposure of 0.0002 μg/kg body weight per day.^26^ Notably, one sample (sample #64) exceeded this limit by more than 50 times and another four samples exceeded the limit by more than three times. Although BPA levels in mask materials are not yet regulated by authorities, previous studies have shown that even at low concentrations, BPA can adversely affect various body functions.^22,35^ Therefore, these results highlight a previously unrecognized health risk associated with mask-wearing that warrants the attention of both the general public and regulatory agencies.

Among the tested mask samples, the results from analyzing the collected toddler masks were particularly alarming. Specifically, BPA was detected in all of the toddler masks, with one sample showing levels exceeding the European Food Safety Authority (EFSA) BPA limit. The presence of BPA in toddler masks is especially concerning because children have underdeveloped defense and nervous systems. BPA may exert even more adverse effects on children than on adults.^36^ In addition to its estrogen-like endocrine-disrupting effects, previous studies have shown that BPA significantly impacts brain development and function.^37−39^

### Mask-to-Landfill Leachate Migration of BPA

Results from previous studies have shown that BPA readily leaches into food and water from food packaging and plastic bottles, respectively.^17−20^ With an estimated of over 81% of face masks ending up in landfills,^11^ we hypothesize that a similar phenomenon may occur with BPA in face masks, potentially releasing it into the environment. This could pose an environmental hazard if landfill leachate improperly contained BPA and leaked into the external environment.

**Figure 4:**
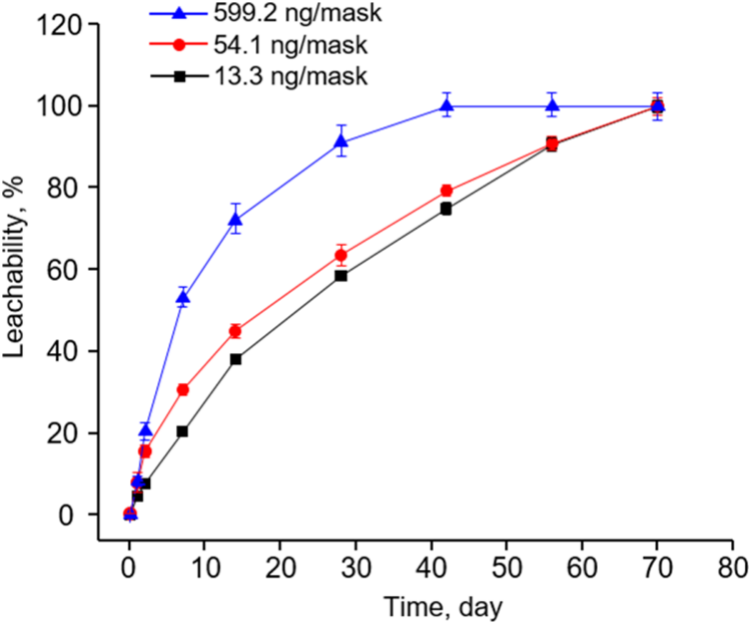
Proportion of mask-borne BPA dissolved into landfill leachate from masks containing different amounts of BPA after being soaked in artificial landfill leachate at 37 °C for different durations of time. The data represent mean ± SD for three independent experiments.

To test this hypothesis, we incubated intact masks with varying BPA contents (599.2 ± 2.1, 54.1 ± 0.2, and 13.3 ± 0.1 ng/mask) in artificial landfill leachate, collecting the leachate at different time intervals for analysis. The results showed a rapid dissolution of BPA into the leachate, with most of the BPA being released within 70 days for BPA-containing masks (Figure 4). Notably, the fastest dissolution occurred in masks with a higher BPA content (599.2 ± 2.1 ng/mask), with complete dissolution achieved within 42 days, probably attributed to the higher concentration gradient that increased the rate of dissolution of BPA into the landfill leachate.^40^

**Figure 5:**
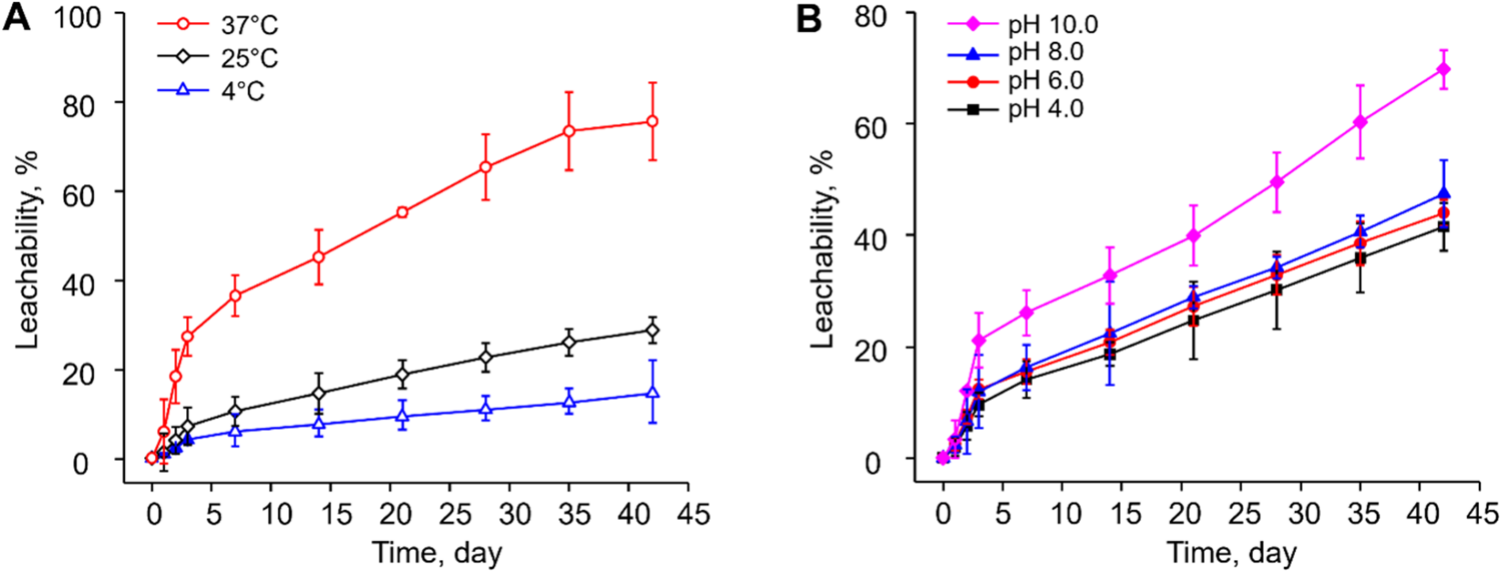
Effects of (A) temperature and (B) acidity of the landfill leachates on the proportion of mask-borne BPA dissolved into landfill leachate from masks containing 343.0 ± 20.9 ng of BPA after soaking in artificial landfill leachate for different duration of time. The data represent mean ± SD for three independent experiments.

Using the same set of mask samples, we investigated the impact of environmental factors on BPA release. The results demonstrated a facile temperature- and pH-dependent release of BPA into the leachate with higher temperature and pH accelerating the dissolution process (Figure 5). While increasing the pH from 4.0 to 8.0 slightly increases the fraction of BPA in its ionized form, facilitating its dissolution,^41,42^ it is worth mentioning that at pH 10.0, which is near its p*K*_a_ (9.6),^41^ the ionizability and, consequently, the leachability of BPA increased significantly. Similarly, a higher BPA leachability was observed in old landfill leachate (Figure S4), in which the pH was higher than that of the young leachate used for the whole experiment.^41,43,44^

Based on the analysis of 85 mask samples, it is estimated that each face mask contains an average of 71.8 ng of BPA. Given the daily consumption of approximately 4 billion masks,^2^ this leads to over 0.3 tons of BPA being released into the environment from used face masks throughout the pandemic, which lasted approximately 3 years and 3 months.^45^ It is important to note that this estimate does not include disposable medical isolation gowns made from similar materials, which were also extensively used during this period.

Although landfill leachate in developed countries is typically contained for further chemical treatment, facilities in developing countries are often inadequately equipped and poorly maintained, which may lead to the release of leachate into the environment. Given the vast number of face masks used during the pandemic, the release of mask-borne BPA into the environment may represent a previously unrecognized environmental hazard.

## Conclusions

In this study, we report for the first time the widespread presence of BPA, a well-known estrogen-like endocrine disruptor, in disposable face masks at concentrations as high as 2 μg/mask. Our results indicate that BPA from the masks can be readily transferred to sweat and landfill leachate and potentially via placental transport and breastfeeding to infants,^46^ revealing previously unrecognized risks of human exposure and environmental hazards. With extensive face mask usage during the past years of the pandemic and their increasing usage by the general public even in the postpandemic years, these findings highlight the need for increased awareness among mask wearers and attention from regulatory agencies.

## Supporting Information Available

Composition of artificial sweat and artificial landfill leachate; method accuracy, precision, and detection limits; calibration parameters; amount of BPA in inner layers and ear loops of masks with the estimated daily intakes; chromatograms from LC–MS/MS analysis of TBBPA and BPA in typical mask samples; amount of BPA in different mask layers and the ear loop; and effect of the age of leachate on the leachability of BPA into landfill leachate (PDF)

## Abbreviations

BPA: bisphenol A
TBBPA: tetrabromobisphenol A
LC–MS/MS: liquid chromatography-tandem mass spectrometry
