# Supplementary material for: Bisphenol A in Disposable Face Masks: A Novel Human Exposure Pathway and Impact on the Aquatic Environment
Source: Chem Res Toxicol. 2025 Feb 3;38(2):347–52. doi: 10.1021/acs.chemrestox.4c00535 (PMC11837208; doi:10.1021/acs.chemrestox.4c00535)
Supplement: Supplementary file 1 — tx4c00535_si_001.pdf [file tx4c00535_si_001.pdf]

Supporting Information

Bisphenol A in Disposable Face Masks: A Novel Human Exposure Pathway and Impact on the Aquatic Environment

Hei-Tak Tse, Chun-Kit Au, and Wan Chan *

Department of Chemistry, The Hong Kong University of Science and Technology, Clear Water Bay, Kowloon, Hong Kong.

E-mail: chanwan@ust.hk; Phone: +852 2358-7370; Fax: +852 2358-1594.

TABLE OF CONTENTS

Table S1. Composition of artificial sweat and artificial landfill leachate. (Page S3)

Table S2. Calibration parameters for LC−MS/MS analysis of bisphenols. (Page S4)

Table S3. Method accuracy and precision of bisphenol analysis. (Page S5)

Table S4. Amount of BPA in inner layer and ear loop of masks, and the estimated ng BPA/kg body weight exposed. (Page S6 – S8)

Figure S1. Typical LC−MS/MS chromatogram of TBBPA in standard solution and mask sample. (Page S9)

Figure S2. Typical LC−MS/MS chromatogram of BPA in standard solution and mask sample. (Page S10)

Figure S3. Amount of BPA and its associated analogues in different layers and the ear loop in the disposable face masks. (Page S11)

Figure S4. Effect of ages of landfill leachate on dissolution of BPA in artificial landfill leachate. (Page S12)

Table S1. Composition of Artificial Sweat and Artificial Landfill Leachate.

Composition (g/L)	Artificial sweat	Artificial young leachate	Artificial old leachate
L-Histidine	0.50	--	--
NaCl	5.00	--	--
$NaH_2PO_4·2H_2O$	2.20	--	--
Sodium acetate	--	0.10	--
Sodium propionate	--	0.0050	--
Sodium humate	--	0.050	0.25
NH_4Cl	--	0.38	0.19
$CaCl_2·2H_2O$	--	0.11	0.037
$MgCl_2·2H_2O$	--	0.084	0.084
$CdCl_2·2.5H_2O$	--	0.0061	0.0020
$NiCl_2·6H_2O$	--	0.012	0.0040
$ZnSO_4·7H_2O$	--	0.013	0.0044
$MnCl_2·4H_2O$	--	0.011	0.0036
$K_2Cr_2O_7$	--	0.017	0.0057
$FeCl_3·6H_2O$	--	0.015	0.0048
$Pb(NO_3)_2$	--	0.0065	0.0022

Table S2. Calibration Parameters for the LC−MS/MS Analysis of Bisphenols in Mask Samples.

	BPA	BPF	BPC	BPAP	BPAF
Linear range, µg/L	0.5 – 50	0.5 – 50	0.5 – 50	0.5 – 50	0.5 – 50
Slope	0.97	0.42	0.17	2.50	4.29
Intercept	0.022	-0.004	0.0001	0.071	0.002
r^2	0.9998	0.9997	0.9997	0.9994	0.9998
MDL, pg/injection [a]	1.0	0.5	1.3	2.0	1.0
MDL, ng/g [b]	3.0	1.5	3.9	6.0	3.0

[a] MDL expressed as the minimum amount of bisphenols that generated an analytical signal three times the noise.

[b] The MDL is the sample extract based on 100 mg of mask sample.

Table S3. Method Accuracy and Precision of the Developed LC−MS/MS Method for the Analysis of BPA and its Associated Analogues in Mask Samples.

	Accuracy			Precision	
Analyte	Spiked, ng/mask	Found, ng/mask [a]	Recovery, %	Interday, %RSD [a]	Intraday, %RSD [a]
BPA	15	14.7 ± 1.1	97.8 ± 7.3	8.2	7.5
	150	142.0 ± 2.2	94.7 ± 1.4	3.8	1.5
	1500	1320 ± 23.8	88.8 ± 1.6	4.2	1.7
BPF	3	2.67 ± 0.03	88.7 ± 1.0	2.7	1.6
	15	15.0 ± 0.60	100.1 ± 4.0	4.7	4.0
	30	28.4 ± 0.51	94.5 ± 1.7	1.6	1.8
BPC	3	2.80 ± 0.12	92.9 ± 3.7	6.7	4.0
	15	13.3 ± 0.48	88.4 ± 3.3	4.5	3.7
	30	26.4 ± 2.55	87.9 ± 8.5	7.4	9.7
BPAP	1.5	1.32 ± 0.06	87.2 ± 4.3	8.1	5.0
	3	2.49 ± 0.06	82.7 ± 2.0	5.8	2.5
	15	13.6 ± 0.72	90.9 ± 4.8	5.2	5.3
BPAF	1.5	1.59 ± 0.06	106.8 ± 2.8	6.3	2.6
	3	2.76 ± 0.03	92.4 ± 1.3	3.1	1.4
	15	16.4 ± 0.15	109.4 ± 1.0	3.7	0.9

[a] $n = 7$

Table S4. Amount of BPA in Inner Layer and Ear Loop of Masks, and the Estimated ng BPA/kg Body Weight Exposed.

ID	Inner, ng/g	Ear loop, ng/g	EDI, ng BPA/kg body weight
1	0.89	0.85	0.00
2	0.89	1.74	0.01
3	0.89	8.38	0.03
4	5.20	5.87	0.03
5	3.42	5.21	0.02
6	0.00	17.46	0.07
7	0.00	4.56	0.02
8	0.89	0.85	0.00
9	6.50	5.44	0.04
10	0.00	2.40	0.01
11	2.04	0.85	0.00
12	0.89	0.00	0.00
13	0.89	11.35	0.06
14	4.12	0.85	0.01
15	0.89	0.85	0.00
16	3.07	0.85	0.01
17	2.87	1.99	0.01
18	0.00	0.85	0.00
19	0.89	0.85	0.01
20	0.89	0.85	0.00
21	4.40	24.38	0.12
22	5.35	4.77	0.04
23	3.31	0.85	0.01
24	0.00	4.55	0.02
25	0.00	6.55	0.03
26	0.00	6.29	0.03
27	0.00	6.20	0.03
28	0.89	3.19	0.01
29	0.89	0.85	0.00
30	0.89	4.84	0.02
31	0.89	34.37	0.12
32	986.41	256.11	2.13
33	0.89	10.38	0.04

34	6.31	3.49	0.02
35	0.89	0.85	0.00
36	0.89	2.97	0.01
37	0.89	3.15	0.02
38	2.62	5.64	0.03
39	0.89	4.96	0.04
40	3.68	2.08	0.01
41	0.00	27.74	0.14
42	4.62	3.23	0.02
43	0.00	0.00	0.00
44	1354.67	12.02	1.64
45	270.93	205.75	0.36
46	503.62	329.85	0.63
47	0.00	0.85	0.00
48	0.00	14.00	0.05
49	0.89	8.02	0.07
50	0.00	1.86	0.02
51	0.00	9.52	0.09
52	0.89	0.85	0.01
53	1.95	2.03	0.02
54	0.00	7.58	0.07
55	0.00	1.47	0.01
56	0.00	0.00	0.00
57	0.00	0.00	0.00
58	3.29	4.21	0.04
59	0.89	0.00	0.01
60	0.89	6.53	0.06
61	0.00	1.34	0.01
62	0.00	17.42	0.16
63	3.18	1123.03	10.05
64	0.89	0.85	0.01
65	0.00	0.85	0.01
66	0.00	64.75	0.58
67	0.00	19.58	0.18
68	0.00	9.50	0.08
69	11.07	7.42	0.09
70	8.99	0.00	0.02

71	1.46	5.59	0.05
72	0.00	3.78	0.03
73	0.00	9.42	0.04
74	1.60	5.22	0.21
75	0.89	0.85	0.01
76	0.89	1.70	0.04
77	0.00	0.85	0.01
78	0.00	2.14	0.05
79	2.05	2.22	0.05
80	0.89	0.85	0.05
81	0.89	0.85	0.04
82	2.62	11.44	0.27
83	0.89	2.55	0.07
84	2.48	6.27	0.17
85	1.95	2.56	0.07

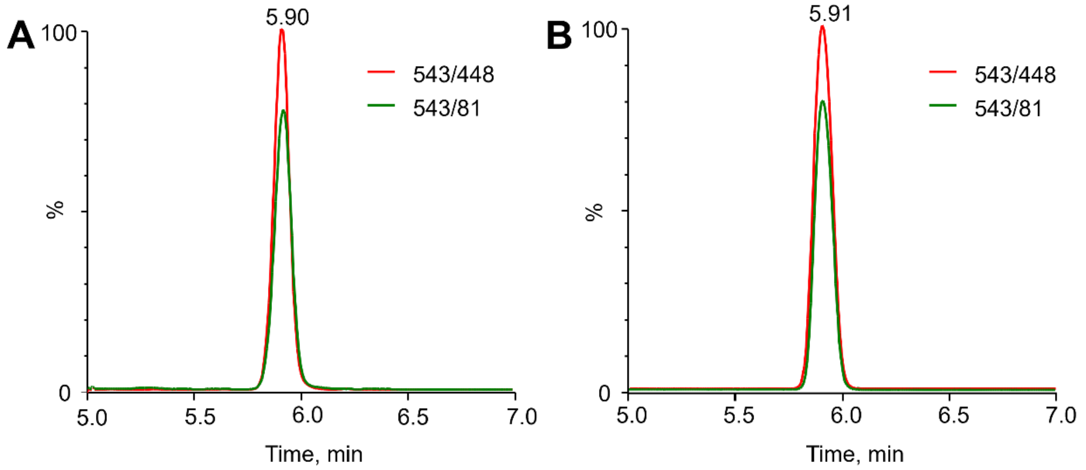

Figure S1. Typical LC–MS/MS chromatograms from MRM monitoring of TBBPA in (A) standard solution and in (B) a typical mask sample. Analysis performed on a Waters TQ-XS HPLC–MS/MS system using a lunar C18 column.

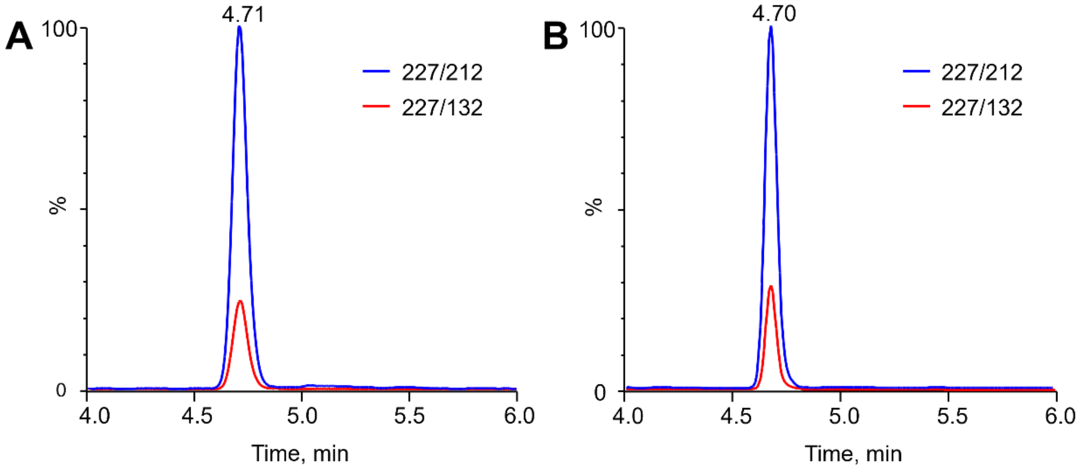

Figure S2. Typical LC–MS/MS chromatograms from MRM monitoring of BPA in (A) standard solution and in (B) a mask sample.

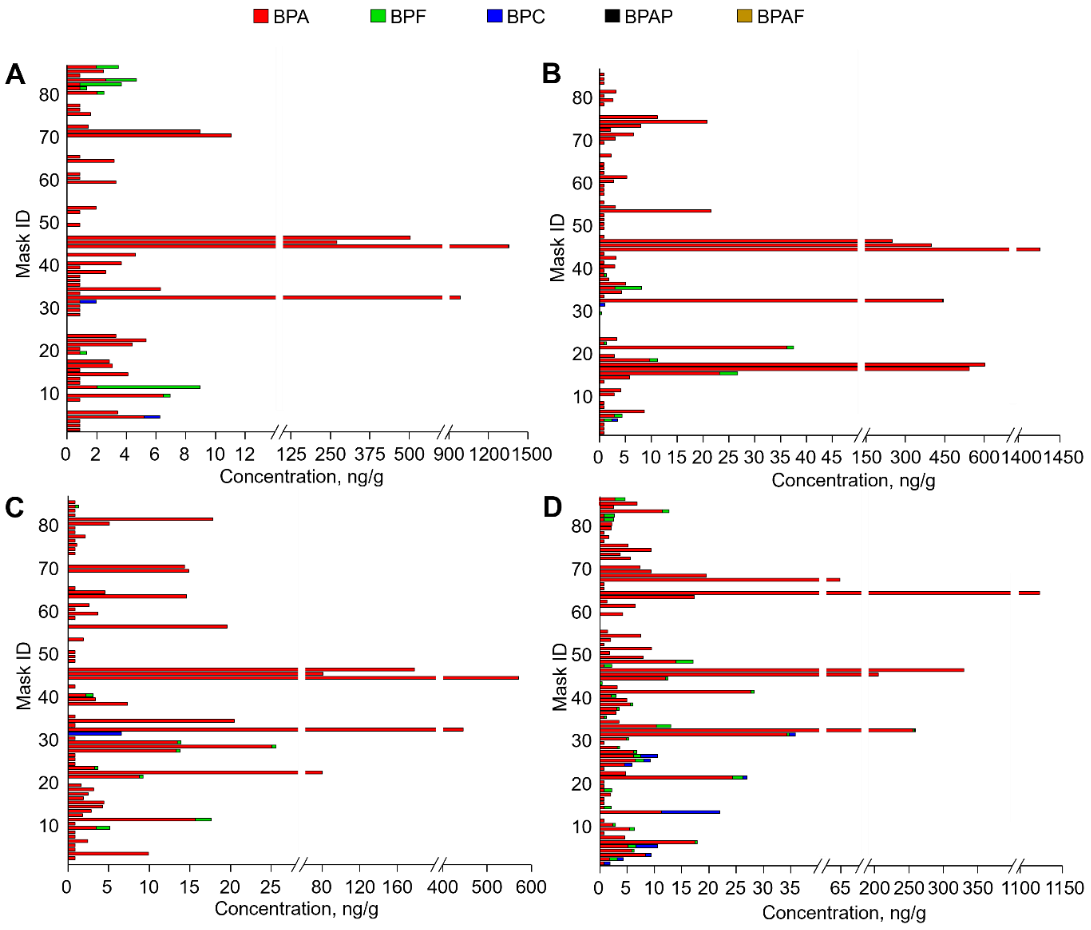

Figure S3. Amount of BPA and its associated analogues in (A) inner layer, (B) middle layer, (C) outer layer, and (D) ear loop in the disposable face masks.

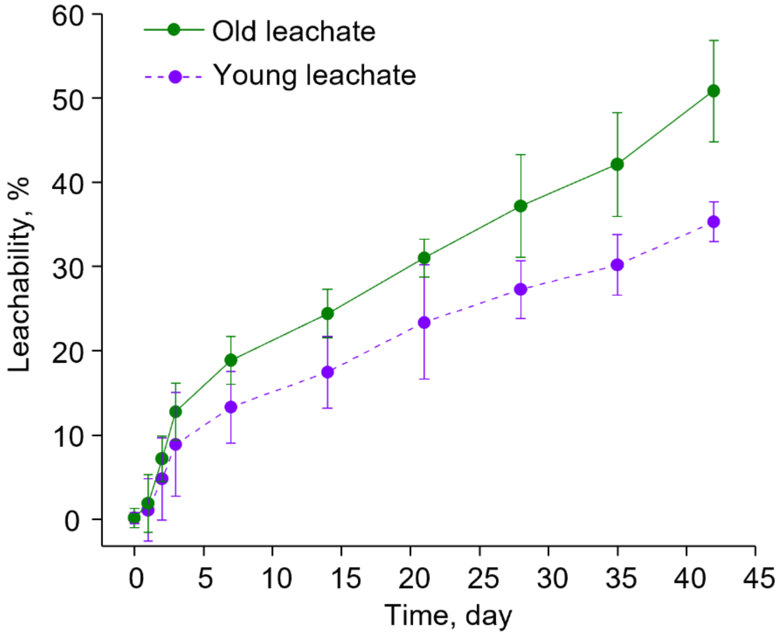

Figure S4. Effect of ages of landfill leachate on dissolution of BPA in artificial landfill leachate. The data represent mean ± SD for three independent experiments.
